# Depletion of Akt1 and Akt2 Impairs the Repair of Radiation-Induced DNA Double Strand Breaks via Homologous Recombination

**DOI:** 10.3390/ijms20246316

**Published:** 2019-12-14

**Authors:** Tahereh Mohammadian Gol, H. Peter Rodemann, Klaus Dittmann

**Affiliations:** 1 Division of Radiobiology and Molecular Environmental Research, Department of Radiation Oncology, University of Tübingen, Röntgenweg 11, 72076 Tübingen, Germany; tahereh.mohammadian-gol@student.uni-tuebingen.de; 2 DKFZ Partner Site Tübingen, German Cancer Consortium, German Cancer Research Center, 69120 Heidelberg, Germany

**Keywords:** DSBs, HRR, Akt isoforms, human colorectal cancer

## Abstract

Homologous recombination repair (HRR), non-homologous end-joining (NHEJ) and alternative NHEJ are major pathways that are utilized by cells for processing DNA double strand breaks (DNA-DSBs); their function plays an important role in the radiation resistance of tumor cells. Conflicting data exist regarding the role of Akt in homologous recombination (HR), i.e., the regulation of Rad51 as a major protein of this pathway. This study was designed to investigate the specific involvement of Akt isoforms in HRR. HCT116 colon cancer cells with stable AKT-knock-out and siRNA-mediated AKT-knockdown phenotypes were used to investigate the role of Akt1 and Akt2 isoforms in HR. The results clearly demonstrated that HCT116 AKT1-KO and AKT2-KO cells have a significantly reduced Rad51 foci formation 6 h post irradiation versus parental cells. Depletion of Akt1 and Akt2 protein levels as well as inhibition of Akt kinase activity resulted in an increased number of residual-γH2AX in CENP-F positive cells mainly representing the S and G2 phase cells. Furthermore, inhibition of NHEJ and HR using DNA-PK and Rad51 antagonists resulted in stronger radiosensitivity of AKT1 and AKT2 knockout cells versus wild type cells. These data collectively show that both Akt1 and Akt2 are involved in DSBs repair through HRR.

## 1. Introduction

DNA double-strand breaks (DSBs) are the most lethal type of DNA lesions that can be generated via ionizing radiation [1,2]. To protect the genome from these potentially lethal lesions, cells employ two main DNA repair mechanisms: the classical non-homologous end joining (C-NHEJ) and the homologous recombination (HR). Here, C-NHEJ is the major pathway, however there is an error-prone mechanism to repair DNA-DSBs in which the DNA-dependent protein kinase catalytic subunit (DNA-PKcs) plays a critical role. Binding DNA-PKcs to Ku80 at the site of damage and its activation is needed to activate end processing enzymes. This is followed by rejoining of DNA ends via the XRCC4-ligase IV complex and XLF [3,4]. A DNA-PKcs independent backup pathway known as alternative-NHEJ (A-NHEJ) can be used to remove DNA-DSBs in the absence of functional DNA-PKcs-dependent NHEJ [5].

Both of the error-prone NHEJ repair pathways are active throughout the entire cell cycle (except M phase). In contrast, HR is an error-free mechanism that acts only in the late S and G2 phase because the HR pathway requires a homologous chromatid for repair. HR is initiated via DSB resection by the MRN complex (MRE11, NBS1, Rad50) and produces a short patch of single-stranded DNA. Replication protein A (RPA) covers the single stranded DNA and prevents the formation of secondary structures. In a subsequent step, recombinase Rad51 replaces RPA via recombination mediators such as BRCA2. Rad51 then invades the sister chromatid and forms a D-loop. After replacement of the damaged site of DNA by the homologous strand, HR can be completed via different pathways including synthesis-dependent strand-annealing (SDSA), the classical double-strand break repair (DSBR), and break-induced replication (BIR) [6,7,8,9].

The serine/threonine protein kinase B (PKB/Akt) is a critical downstream factor of PI3K pathway and is a frequently activated oncoprotein. Three isoforms of Akt exist with high homology, i.e., Akt1, Akt2, and Akt3; these Akt isoforms play important roles in cellular processes and their dysfunction is documented for a variety of human cancers [10,11]. Accumulating evidence indicates a regulatory role of Akt isoforms—especially Akt1 and Akt3 in NHEJ [12,13,14,15]. Akt1 has also been reported to be potentially involved in HR-dependent repair processes [16], however these data conflict with other reports [16,17,18].

Mueck et al. [16] indicated that Akt1 promotes HRR by influencing Rad51 protein levels and foci formation. Jia et al. [17] reported a negative role of Akt on HRR via the CHK1-Rad51 pathway in BRCA1-deficient cells. Likewise, Plo et al. [18] postulated that Akt1 inhibits homologous recombination by inducing cytoplasmic retention of BRCA1 and RAD51 as detected with immuno-fluorescence. Thus, the current functional role of Akt and its isoforms in HR is not clearly understood and the existing data are conflicting [16,17,18].

This work is based on knock-down and knock-out strategies—we more specifically characterized the importance of Akt isoforms Akt1 and Akt2 for homologous recombination repair of DSBs in human colorectal cancer cells in vitro. In contrast to Mueck et al. [16], Jia et al. [17], and Plo et al. [18], the HR analyses were performed primarily in cells positive for the centromeric protein CENP-F. This is a clear indicator of the late S- as well as G2-phase cells. We found that inhibition of C-NHEJ and A-NHEJ repair results in a significantly increased number of residual γ-H2AX foci in CENP-F positive cells after radiation, indicating a deficient HRR in AKT1- and AKT2- knock out cells. Thus, our data demonstrate that both Akt1 and Akt2 are involved in double strand break repair via the HRR pathway in the HCT116 human colorectal cancer cell line.

## 2. Results

### 2.1. Importance of Akt1 and Akt2 for HR Repair of DSBs

Akt exerts an important regulatory role in the repair process of DSBs via the NHEJ pathway [12,13,15,19,20]. Likewise, our laboratory presented the first evidence for a potential involvement of Akt in HR-repair [16]. Therefore, we addressed the question of the potential regulatory influence and impact of Akt isoforms Akt1 and Akt2 on the functionality of HR. To do this, we used human colorectal carcinoma cells with inactivated AKT1 and AKT2 genes. The number of γ-H2AX foci was monitored 24 h after 4Gy radiation in cells positive for the centromeric protein CENP-F. This number was an indicator of non-repaired DNA damage and indicates cells present in the late S and G2 phase of the cell cycle.

**Figure 1:**
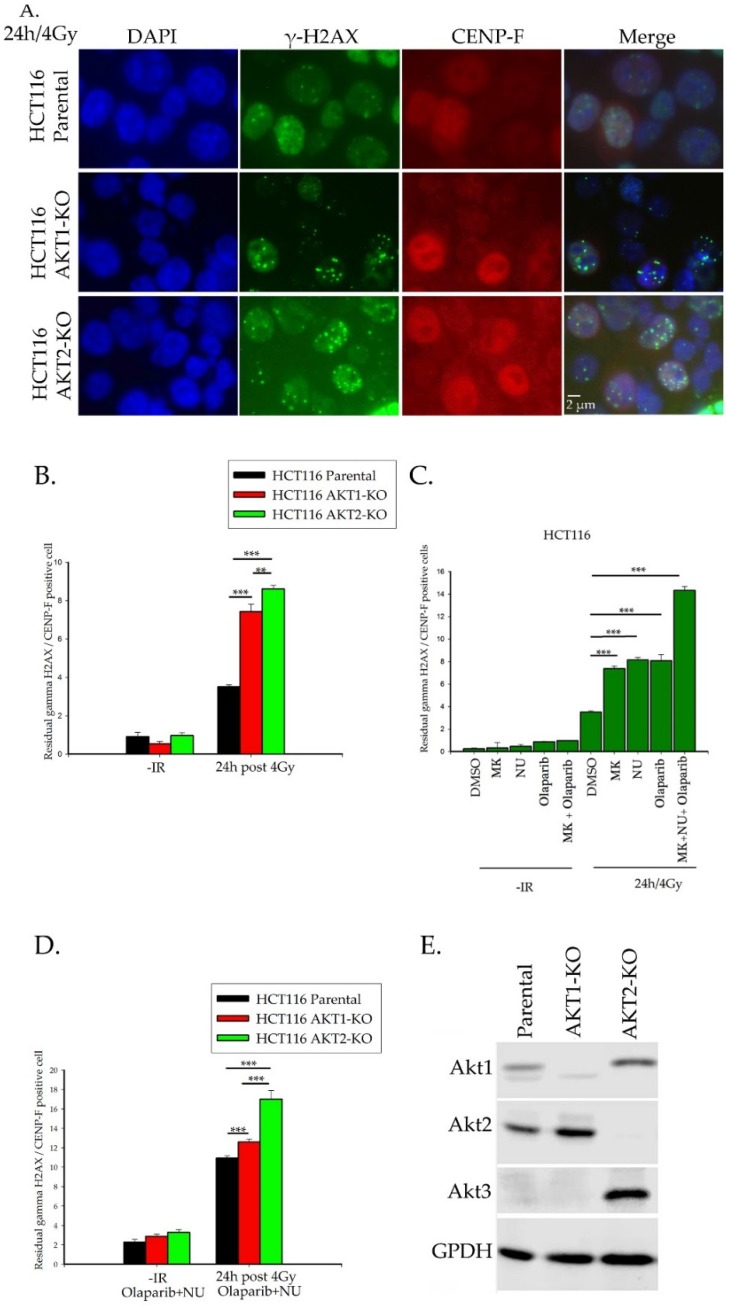
AKT depletion leads to increased residual DNA-DSB in irradiated CENP-F positive cells. (**A**) Immunofluorescence analysis was performed as described in the material and methods. The γ-H2AX foci (green) 24 h after 4 Gy in CENP-F positive cells (red). (**B**) The number of γ-H2AX foci was analyzed 24 h after 4 Gy radiation. (**C**) The HCT116 parental cells were treated with 1 µM of inhibitors (MK2206, NU7441, and Olaparib) for 2 h and irradiated with 4 Gy. The number of γ-H2AX foci were counted 24 h after irradiation. (**D**) The HCT116 parental and knockout cells were irradiated 2 h after treatment with a combination of NU7441 and Olaparib (1 µM each). The data represent the mean ± SEM of three independent experiments and a total of at least 300 nuclei per condition (*p* < 0.05, ** *p* < 0.01, and *** *p* < 0.001, Student’s *t*-test). (**E**) The Akt isoform protein levels were quantified by western blotting in the HCT116 parental and knockout cells (**B**).

Figure 1A,B show the significant increase in the amount of residual γ-H2AX foci in both irradiated AKT1- and AKT2-knock out cells. Although the induction of DNA-DSBs in parental and knockout cells was very similar (Figure S1), the significant increase of residual γ-H2AX foci in knockout cells clearly indicates a role of Akt1 and Akt2 for DNA-DSB-repair in late S and G2-phase cells. Likewise, inhibition of Akt-kinase activity by the pan-AKT inhibitor MK2206 also resulted in a significant increase of residual γ-H2AX foci in CENP-F positive cells, again indicating an impaired DNA-DSB repair process (Figure 1C).

To more specifically assess the involvement of HR in Figure 1A,B, we blocked C-NHEJ and A-NHEJ pathways in parental HCT116 via the DNA-PK inhibitor NU7441, the PARP inhibitor Olaparip, and the MK2206 inhibitor. Treatment with either DNA-PK, PARP inhibitors, or MK2206 prior to irradiation of cells with 4 Gy significantly increased the number of γ-H2AX foci. The combination of all three inhibitors led to a nearly two-fold increase in the number of γ-H2AX foci versus single treatment conditions (Figure 1C).

Combination therapy with NU7441 and Olaparib significantly stimulated the number of residual γ-H2AX foci in irradiated AKT1 and AKT2 knockout cells versus irradiated parental control cells (Figure 1D). However, this effect was significantly more pronounced in AKT2-KO than in AKT-1 KO cells (Figure 1D). As demonstrated in Figure 1E, the AKT3 isoform is neither expressed in the parental HCT116 nor in AKT-1 KO cells of this cell line but was detectable in AKT2-KO cells. This result confirms data reported by Ericson et al., [21] indicating that AKT3 is inactive in HCT116 parental and HCT116 AKT-1- KO cells. However, the data in Figure 1D suggests that the induction of AKT3-expression in AKT2-KO cells cannot compensate for the effect of AKT2 depletion on DNA-DSB repair in CENP-F positive cells. These data indicate that both Akt1 and Akt2 play an important role in the homologous recombination repair pathway.

### 2.2. Akt Mediates Radioresistance Through Stimulated Homologous Recombination

**Figure 2:**
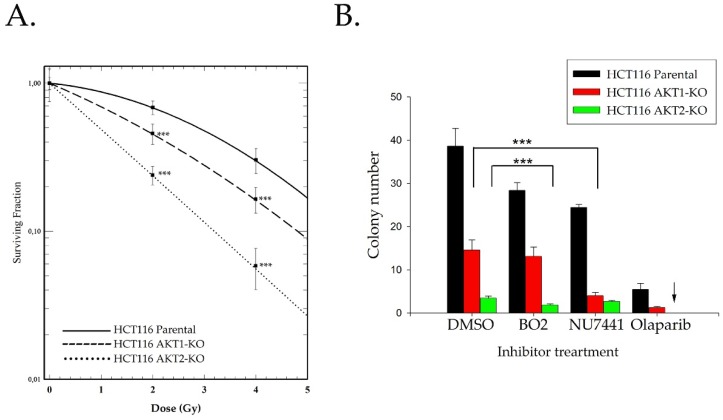
Clonogenic cell survival of irradiated HTC116 parental and AKT1-KO and AKT2-KO cells. Clonogenic assays were performed as described in the Materials and Methods. (**A**) HCT116 parental, AKT1-KO, and AKT2-KO cells irradiated with 0, 2, and 4 Gy. Data points represent the mean surviving fractions (SF) ± the standard deviation (SD) of three independent experiments (*n* = 18; and *** *p* < 0.001, Student’s *t*-test). (**B**) All three cell lines were treated with 1 µM of the indicated inhibitors 2 h before radiation exposure 2 Gy. Bars represent the average number of colonies formed when 350 cells were seeded for the different treatment conditions (*n* = 18; ** *p* < 0.01, and *** *p* < 0.001, Student’s *t*-test).

The repair efficacy data shown in Figure 1 was used to analyze the clonogenic post-irradiation cell survival of HCT116 wild type and AKT-deficient cells. Figure 2 demonstrates that clonogenic cell survival after radiation exposure was significantly impaired in AKT1 and AKT2 knockout cells. However, the AKT2-KO cells showed a significantly stronger effect than AKT1-KO cells (Figure 2A). Moreover, when AKT1-KO cells were treated with the DNA-PK inhibitor NU7441 and 2 Gy irradiation, the AKT1-KO cells responded with a stronger sensitivity to NHEJ inhibition than parental cells. Blocking HR via the Rad51 inhibitor BO2 resulted in a significant reduction in the colony formation of AKT2-KO cells versus parental cells (Figure 2B). As indicated in supplementary Figure S2, BO2 is able to block HR by inhibiting Rad51 foci formation in the nucleus.

Olaparib is a PARP inhibitor that is approved for clinical use in HR-deficient tumors such as BRCA-deficient tumors. Nonfunctional HR enhances the sensitivity of cells to Olaparib [22,23]. We observed that AKT1-KO cells are more sensitive to PARP inhibition than parental cells. The sensitivity of AKT-KO cells to Olaparib was extremely elevated when AKT2-KO cells were tested. As indicated by the arrow in Figure 2B, colony formation of AKT2-KO cells under Olaparib treatment was completely inhibited.

### 2.3. Akt1 and Akt2 Affect the Regulation of Rad51 Translocation and its Loading to the Site of Damage

#### 2.3.1. Rad51 Foci Formation and Nuclear Translocation

**Figure 3:**
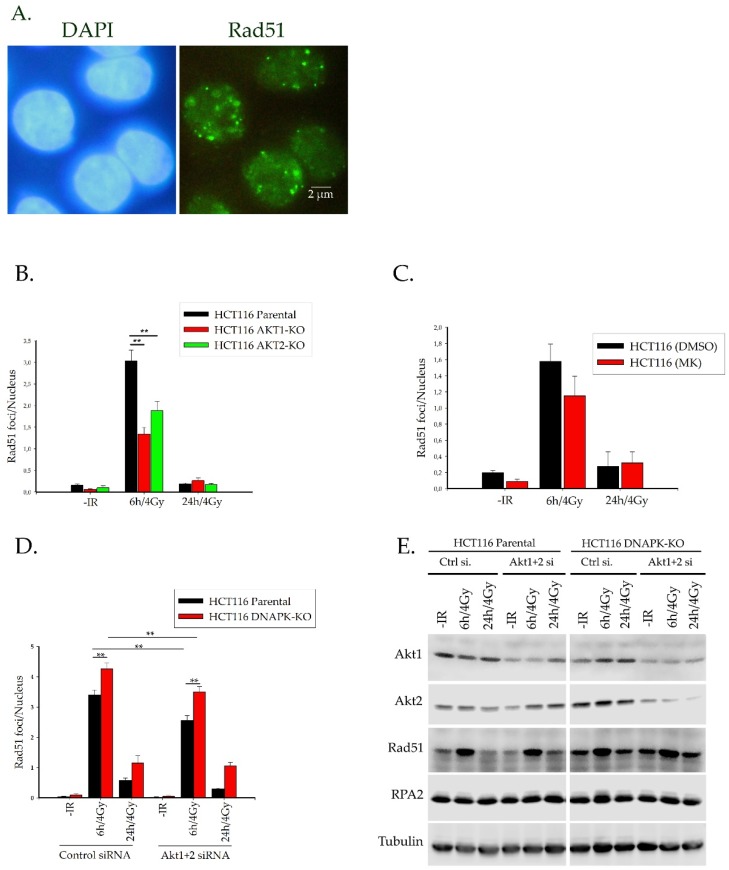
Determination of Rad51 foci formation and protein levels in HCT116 parental and AKT1/AKT2 KO cells. (**A**) Immunofluorescence analysis was performed as described in the Materials and Methods. Rad51 foci (green) in sub-confluent cells (blue). (**B**) Rad51 foci number per nucleus were analyzed after immunofluorescence staining of HCT116 parental, AKT1-KO, and AKT2-KO (**C**) after 2 h MK2206 (1 µM) treatment as well as after irradiation. (**D**) HCT116 parental and DNAPK-KO cells were transfected with AKT1-siRNA, AKT2-siRNA, and control-siRNA. The number of Rad51 foci were counted at the indicated time points after 4 Gy. Bars represent the mean number of foci/cell ± SEM from at least three independent experiments. (**E**) siRNA transfection efficacy was analyzed via a western blot (** *p* < 0.01, Student’s *t*-test).

A previous study from our laboratory [16] showed that AKT1-siRNA knockdown in the non-small cell lung cancer cell line A549 significantly reduced both Rad51 protein levels and foci formation. To confirm these previous findings, we analyzed Rad51 foci formation in HCT116 AKT1 and AKT2 knock out cells. Figure 3B shows that the number of Rad51 foci 6 h after exposure to irradiation IR was significantly repressed versus parental HCT116 control cells. Interestingly, however, a 2 h treatment with the Akt-inhibitor MK2206 prior to radiation exposure resulted in only a slight but non-significant decrease in Rad51 foci 6 h post-irradiation (Figure 3C). To assess whether a non-functional NHEJ pathway can increase Rad51 foci formation after radiation exposure, we performed a siRNA-mediated dual knockdown of AKT1 and AKT2 in HCT116 DNA-PK-deficient cells. As demonstrated in Figure 3D in the absence of functional cNHEJ repair, the number of Rad51 foci enhanced about 20% versus the cNHEJ-proficient cells. This is due to a compensatory stimulation of HR. This idea is supported by Western blot data indicating that NHEJ-deficient cells present up-regulated expression of Rad51 protein (Figure 3E). Single knockdown of Akt1 and Akt2 led to a decrease of Rad51 foci formation in parental and DNAPK-KO cells. The decrease of Rad51 foci formation was even stronger after the knockdown of Akt2 in DNAPK-KO cells (Figure S3). However, siRNA-mediated downregulation of both Akt isoforms resulted in a significant reduction of Rad51 foci formation in both NHEJ-proficient and deficient cells (Figure 3D). These data clearly indicate that Akt depletion significantly impairs Rad51 loading to DNA-DSBs, which as a consequence, results in an impaired HR repair process.

**Figure 4:**
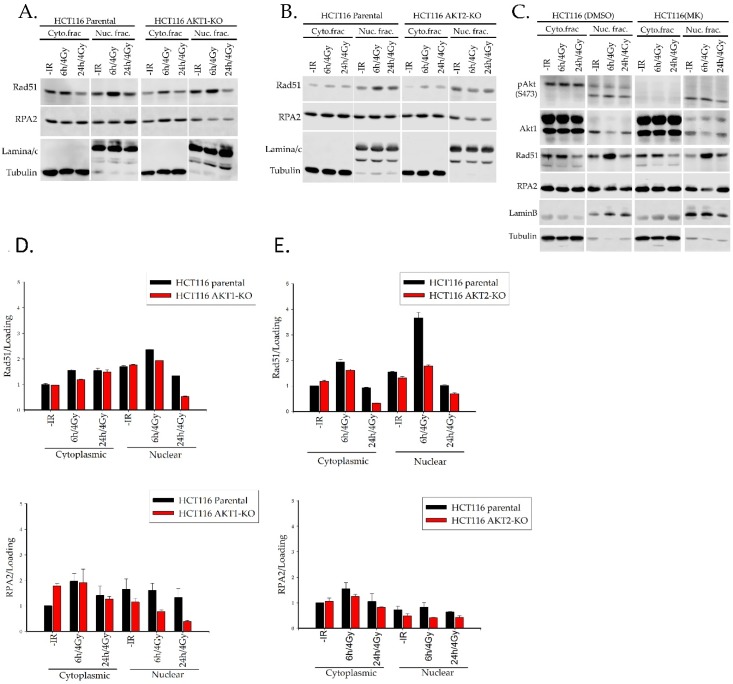
Akt1 and Akt2 are involved in the translocation of Rad51 to the nucleus. (**A**,**B**) HCT116 parental, AKT1-KO, and AKT2-KO cells were irradiated. Six and 24 h later, the cytoplasmic and nuclear fractions were prepared. (**C**) MK2206 (1 µM)-treated and nontreated HCT116 parental cells were irradiated and the cytoplasmic and nuclear fractions were prepared 6 and 24 h later. (**D**,**E**) Rad51 and RPA2 protein levels were determined by western blotting. Tubulin and lamin were used as cytoplasmic and nuclear markers, respectively. Densitometry is based on the mean ± SEM of three independent experiments.

We further asked whether the reduced Rad51 foci formation may be due to an impaired translocation of Rad51 to the nucleus. To address this question, nuclear/cytoplasmic fractionation was performed for HCT116 cells after exposure to 4 Gy. Rad51 translocation was then analyzed via western blotting. Figure 4A,B show that the levels of nuclear Rad51 6 h and 24 h after radiation were markedly reduced both in AKT1-KO and AKT2-KO cells. However, these results indicated a much stronger effect on AKT2-KO cells. Furthermore, the reduced levels of nuclear Rad51 clearly correlated with a reduced level of nuclear RPA2 protein in AKT1- and AKT2-KO cells versus parental cells (Figure 4D,E). The MK2206 treatment blocked Akt phosphorylation but neither affected Rad51 nor RPA2 nuclear translocation, suggesting that the kinase activity of Akt is most likely not involved in the translocation of RPA2 and Rad51 or in the loading of these proteins to the DNA-DSBs sites (Figure 4C). Likewise, as shown in Figure S4, the nuclear translocation of Rad51 and RPA was not affected by the pretreatment of HCT116 DNAPK-KO cells with MK2206.

#### 2.3.2. Rad51 Phosphorylation

**Figure 5:**
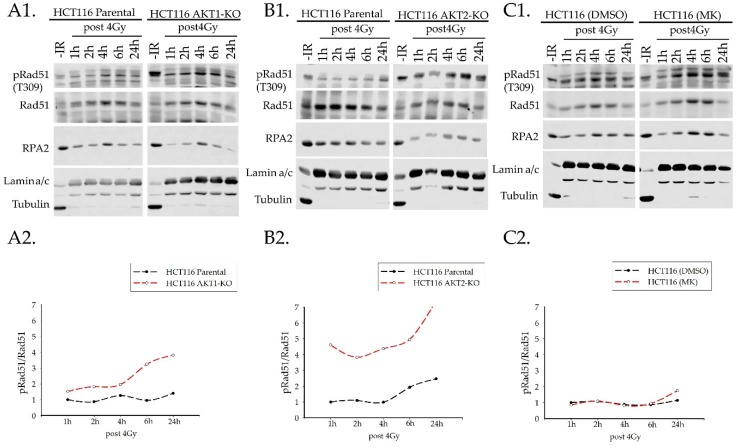
Phosphorylation of Rad51 (T309) after irradiation in AKT1-KO and AKT2-KO cells is higher than the parental cells in the nucleus. Nuclear cytoplasmic fractionations were collected 1, 2, 4, 6, and 24 h after irradiation with 4 Gy as well as non-irradiated cells. (**A1**) HCT116 parental and AKT1-KO, (**B1**) parental and AKT2-KO cells; (**C1**) MK- and DMSO-treated parental HCT116 cells (nuclear fraction). The ratios of nuclear pRad51 (T309)/Rad51 total protein were determined by western blotting and normalized to the level of pRad51 at 1 h for (**A2**) HCT116 parental and AKT1-KO, (**B2**) parental and AKT2-KO cells; (**C2**) MK- and DMSO-treated parental HCT116 cells. The non-irradiated condition was excluded for normalization because of contamination with the cytoplasmic fraction (*n* = 2).

Next, we evaluated the phosphorylation of Rad51 at amino acid residue T309 after radiation of HCT116 parental and AKT1/2-knockout cells. T309 phosphorylation of Rad51 is performed by CHK1 and is required for the formation of nuclear RAD51 foci at the DNA damage site [24]. To test this, we performed nuclear and cytoplasmic fractionation of HCT116 cells at different time points post IR. After irradiation, T309-phosphorylation of Rad51 increased in a time-dependent manner (Figure 5). Compared to parental cells, both AKT1-KO and AKT2-KO cells presented elevated phosphorylation of Rad51 at T309 in the nucleus. Interestingly, the elevated phosphorylation at T309 was more pronounced in AKT2-KO cells (Figure 5A,B). Even at earlier time points (i.e., 30 and 60 min), Rad51 phosphorylation at T309 is increased in the nuclear fraction of both AKT knockout cells (Figure S5). However, after treatment of parental HCT116 cells with the Akt inhibitor, MK2206 phosphorylation of Rad51 at T309 was not altered at least at early time points (i.e., from 1 h to 6 h) after irradiation. Yet, based on the data obtained for the later time point 24 h, after IR we cannot exclude that a small increase in Rad51 T309 phosphorylation is apparent in MK2206 treated cells as shown in Figure 5C.

### 2.4. Interaction of Akt with HR Proteins

**Figure 6:**
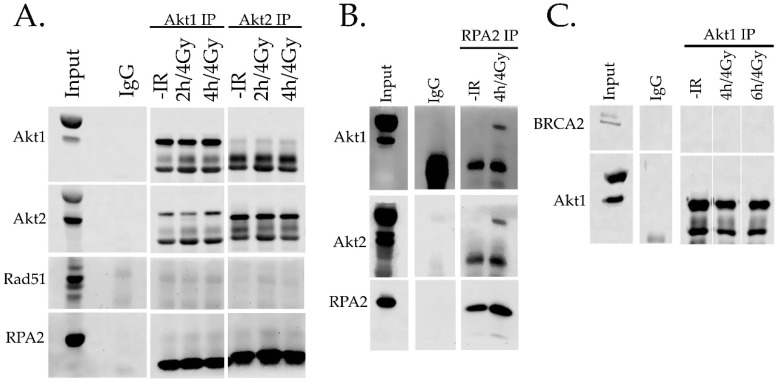
Immunoprecipitation analysis. Akt is not in a complex with BRCA2, RPA2, and Rad51. Immunoprecipitation and co-immunoprecipitation analyses were performed as described in the Materials and Methods. (**A**) Immunoprecipitation of Akt1 and Akt2. (**B**) Immunoprecipitation of RPA2. (**C**) Immunoprecipitation of Akt1.IgG protein immunoprecipitation was used as a control.

The kinase activity of AKT is not involved in the nuclear translocation of Rad51 and RPA (see Figure 4C) and thus, we performed immunoprecipitation experiments to identify a potential direct protein interaction between Akt and Rad51 as well as RPA. Figure 6 shows no clear and stable protein interaction of Akt1 and Akt2 with Rad51 and RPA (Figure 6A). Likewise, immunoprecipitation of Akt did not show any interaction of Akt1 and BRCA2 (Figure 6B,C).

## 3. Discussion

Akt can be activated by the PI3K pathway and plays critical roles in various cellular processes including cell growth, transcriptional regulation, and cell survival [25]. Thus, Akt is an important signal transducer protein for normal as well as tumor cells. In fact, upregulation of Akt-signaling is a feature of various tumor entities [10]. Furthermore, PI3K/Akt signaling and especially Akt through its isoforms Akt1 and Akt3—has been shown to play a regulatory role in the DNA damage response of tumor cells through the NHEJ [12,13,15,26] and potentially HR repair mechanisms [27,28]. Toulany et al., and others [12,15,29] have demonstrated that Akt1 and Akt3 can stimulate DNA-DSB repair through NHEJ, which results in radio-resistance of non-small cell lung cancer cells.

Sahlberg et al., [14] showed that Akt1 and Akt2 isoforms significantly increase the survival of colorectal cancer cells after radiation exposure. Other studies have demonstrated that PI3K pathways are associated with HR by affecting Rad51 recruitment to the site of damage [23,30]. Furthermore, Plo et al. [18] reported that the presence of Akt1 in breast cancers cells resulted in a BRCA1-deficient–like phenotype via cytoplasmic retention of BRCA1 and RAD51. Moreover, Jia et al. [17] showed that the activation of Akt1 in BRCA1-deficient cells impacts the interaction of Chk1 and Rad51 to consequently reduce HR.

On the contrary, Muek et al., [16] showed that siRNA mediated downregulation of Akt1 led to reduced Rad51 protein expression and Rad51-foci formation following radiation exposure of NSCL cancer cells. Likewise, Chang et al. [30] provided evidence that the PI3K/Akt/mTOR pathway is an important regulator of radioresistance of prostate cancer cells through the induction of apoptosis and suppression of autophagy as well as NHEJ and HR repair pathways. The studies performed to date on different in vitro tumor cell lines indicate that Akt1 most likely plays a differential and tumor cell type-specific role in the regulation of HRR.

Thus, in the present study, we aimed to investigate the Akt-dependency of HR more specifically in HCT116 human colorectal cancer cells presenting knockout as well as knockdown phenotypes for the AKT-isoforms AKT1 and AKT2. Our results indicate that in an irradiated CENP-F positive, HR-competent system, both isoforms, i.e., Akt1 and Akt2, are involved in the regulation of the DNA damage response (DDR) executed via HR. This is confirmed by the presence of significantly increased residual γ-H2AX foci in irradiated cells that are positive for the centromeric marker protein CENP-F and downregulated for the isoforms AKT1 and AKT2. In this context, it is important to note that CENP-F-positive cells represent the subpopulation of cells going through the S- and G2-phase of the cell cycle, i.e., the cells that can perform HR after DNA-DSB insults [31].

A previous study from our group [16] showed that Akt1 can stimulate HR in a Rad51-dependent manner. In agreement with this study, we demonstrate here significantly reduced Rad51 foci formation when AKT1 as well as AKT2 isoforms were downregulated alone or in combination with HCT116 colon cancer cells. The results indicate that depletion of AKT1 and AKT2 does decrease Rad51 foci formation and Rad51 protein levels in the nucleus. Interestingly, however, Rad51 phosphorylation at T309 is concurrently increased in the nucleus.

Post-translational modifications of Rad51—especially its phosphorylation—are necessary for its regulation. However, the exact functional importance of the different phosphorylation sites of Rad51 is not yet well understood. For example, phosphorylation on S14 and T13 by Plk1 and CK2 is known to promote Rad51 interaction with NBS1 and its recruitment to the site of damage [32]. Phosphorylation at T315 seems to be involved in Rad51 nucleofilament formation [33]. The phosphorylation at T309 is executed by the kinase activity of Chk1, and Sørensen et al. [24] reported that cells carrying a mutation in this phosphorylation site are sensitive to hydroxyurea. However, this report did not show whether cells presenting this mutation can form Rad51 foci. Likewise, Sørensen et al. [24] did not explain the consequence of this mutation with respect to the accumulation of γ-H2AX foci and efficacy of homologous recombination repair. Marzio et al. [34] showed that in neocarzinostatin (NCS) treated and Rad51 downregulated U2OS cells, mutant Rad51(T309A) could not load on to chromatin as wild type Rad51. They suggested that Rad51 phosphorylation on T309 increases the binding of Rad51 to BRCA2 as well as the stability of Rad51. The authors also noted that this phosphorylation is not sufficient for Rad51-BRCA2 binding and that other modifications of the binding site are required. The function of T309 phosphorylation on Rad51 is currently unclear, but our data indicate that AKT1 and AKT2 depleted cells show increased Rad51 phosphorylation at T309; however, analysis of γ-H2AX foci revealed a reduced repair efficiency.

Reduced appearance of RPA protein in AKT1 and especially in AKT2 knockout cells versus parental cells is indicative of an Akt1- and Akt2-dependent reduction of HRR. This supports our findings of reduced Rad51 protein because it is known that RPA is upstream of Rad51 and triggers binding of Rad51 to the damage site. Binding of RPA to single stranded DNA after the resection process is a prerequisite for coating the damage site with Rad51 [32]. As indicated by the AKT kinase inhibitor data presented here, the effect of Akt on Rad51 as well as RPA regulation seems to be independent of Akt’s kinase activity.

The assembly of RAD51 in the nucleus requires the function of specific mediator proteins such as BRCA2, Rad52, and Rad51 paralogues (Rad51B, Rad51C, Rad51D and XRCC 2, 3). However, the exact mechanism by which these mediators facilitate Rad51 nucleofilament formation is not clear [35]. BRCA2 with highly conserved BRC domains interact with Rad51 and stimulate its nuclear localization and DNA binding ability [36]. To date, however, there is no direct evidence for an interaction of Akt and BRCA2. Despite this and our results showing a role for Akt in Rad51 nuclear accumulation, we could not detect AKT isoforms in direct interactions with BRCA2, RPA, or Rad51. Therefore, the potential involvement of other mediators in this process needs further study.

The role of Akt in the regulation of HRR is further supported by our data presented for the effect of the PARP inhibitor Olaparib. Poly(ADP-ribose) polymerase (PARP) protein is a DNA damage sensor that is important in the repair of DNA single stranded breaks (SSBs) as executed by a base excision repair mechanism (BER). Moreover, PARP is a major component of DSB repair via DNA-PK-independent A-NHEJ [37]. Selective inhibition of PARP leads to radiation sensitivity of HR deficient cells [38,39]. PARP inhibition blocks BER repair of radiation-induced SSBs, which leads to increased DSBs as a result of collapsed replication forks. This effect leads to cell death described as synthetic lethality [40,41,42].

Olaparib is a PARP-inhibitor approved for clinical use in BRCA1- and BRCA2- deficient cancers [23]. This current study indicates that AKT1-KO cells respond to PARP inhibition better than parental cells. More interestingly, AKT2-KO cells do not form colonies after Olaparib treatment. This suggests that the synergistic effect after AKT1 and AKT2 depletion in combination with PARP inhibition may be a consequence of insufficient HRR in the knockout cells.

There is still insufficient information on the role of Akt isoforms in HRR. The studies above [16,17,18] only address the role of Akt1. It is important to investigate the exact function of these isoforms in a cancer cell line-specific manner because cells from different cancer entities differentially present upregulated Akt isoforms. For instance, Rychahou et al., [43] showed that Akt2 is involved in the metastatic process of colorectal cancer, and its upregulation has been reported in colon cancer by Roy et al., [44]. In this context, it is interesting that the results presented here for colon carcinoma HCT116 demonstrate a significantly stronger impact of Akt2 on homologous recombination repair of DSBs than that of Akt1.

There is no detailed mechanistic understanding of the regulatory function of Akt1 and especially, Akt2 in HRR. However, Akt2 may regulate HRR via its described function in glucose metabolism. Akt2 acts as a major regulator of glucose metabolism [45]. In this context, the function of AKT2 is directly correlated with intracellular glucose metabolism and content. Furthermore, ATP-citrate lyase (ACLY) is regulated by ATM and AKT in response to DNA-damage [46]. Nuclear ACLY activity is responsible for the production of nuclear Acetyl-CoA, which is needed for TIP60-driven histone H3 acetylation [47]; histone H3 acetylation is a prerequisite for DNA repair. Indeed, Sivanand et al. [46] reported an Akt-dependent phosphorylation of ACLY at amino acid residue S455 [48] that results in stimulated ACLY activity. This enzymatic activity is associated with an increased acetylation of histone H3 and H4 as well as with the recruitment of BRCA1 to the damage site and initiation of HR [46]. However, these regulatory cascades have not yet been addressed in the context of HR research but do provide a starting point for subsequent detailed analysis.

Taken together, based on the results presented, our study showed that Akt1 and especially Akt2 are involved in the regulation of homologous recombination in human colorectal cancer cells. Therefore, regarding the different functions of Akt isoforms in cancers with different genetic backgrounds and based on our results, it can be argued that blocking specific Akt isoforms might be potentially more effective than pan Akt inhibition in different tumor entities.

## 4. Materials and Methods

### 4.1. Cell lines, Antibodies, and Reagents

The parental colorectal cancer cell line HCT116 (ATCC, CCL-247) was used as well as sublines of HCT116 presenting as genetically knocked out for AKT1 (HCT116-AKT1-KO) or AKT2 (HCT116-AKT2-KO) as well as the HCT116-subline knocked out for DNA-PK (HCT116-DNA-PK-KO). The AKT isoforms knock-out cells were from Horizon (Cambrige, UK) and the DNAPK-KO cells were a gift from Prof. Eric Hendrickson (BMBB Department, University of Minnesota Medical School, Minneapolis, MN, USA). The cells were cultured in RPMI-1640 (Invitrogen, Gibco, Darmstadt, Germany) supplemented with 10% fetal calf serum (FCS) and 1% penicillin-streptomycin. The AKT1-KO and AKT2-KO cells were cultured with 0.3 mg/mL G418 (Invitrogen, Gibco).

Antibodies were purchased from the following sources: P-Akt (Ser-473) (cat. # 4060), Akt1 (cat. # 2967), Akt2 (cat. # 5239) and Akt3 (cat. # 8018) from cell signaling (Frankfurt, Germany); α-tubulin (cat. # CP06) from Calbiochem (Schwalbach, Germany); Rad51 (cat. # ab88572), pRad51 (cat. # ab31769), RPA2 (cat. # ab2175), BRCA2 (cat. # ab27976) and lamin A/C (cat. # ab40567) from Abcam (Cambridge, UK); anti-phospho-Histone H2AX (Ser139) antibody (cat. # 05-636) from Merck Millipore (Darmstadt, Germany); CENP-F (LS-B276) from LSBio (Seattle, WA, USA); and AlexaFluor 488 goat anti-mouse (cat. # A11001) and AlexaFluor 594 donkey anti rabbit (cat. # A32754) from Thermofisher (Darmstadt, Germany).

The Akt inhibitor MK2206 (cat. # S1078), DNA-PK inhibitor NU7441 (cat. # S2638) and the PARP inhibitor Olaparib (cat. # S1060) was purchased from Selleckchem (Houston, TX, USA). Rad51 inhibitor BO2 (cat. # SLM0364) was from Sigma (Taufkirchen, Germany). The siRNA against AKT1 (cat. # M-003000-03) and AKT2 (cat. # M-003001-02) as well as nontargeting siRNA (cat. # D-001810) were purchased from Dharmacon (Lafayette, IN, USA).

### 4.2. Subcellular Fractionation

To identify the post-irradiation localization of proteins of interest, subcellular fractionation experiments were performed to isolate cytoplasmic and nuclear fractions. Therefore, cells were swollen in cytoplasmic lysis buffer (10 mM HEPES, pH 7.9, 10 mM KCl, 0.1 mM EDTA, phosphatase, and protease inhibitors) for 15 min on ice. After adding 5% NP40 and quickly vortexing, the cell lysates were centrifuged at 10,000 RPM for 2 min at 4 °C to sediment the nuclei. The resulting supernatant was separated for cytoplasmic fractionation and the nuclear pellet was washed two times in cytoplasmic lysis buffer and re-suspended in the nuclear lysis buffer (20 mM HEPES, pH 7.9, 400 mM KCl, 1 mM EDTA, 10 mL glycerol, phosphatase, and protease inhibitors). The sample was incubated on ice for 1 h. The nuclear fraction was extracted after sonication and centrifugation (10,000 RPM, 10 min, and 4 °C). Equal amounts of protein were resolved by SDS-PAGE and transferred to a nitrocellulose membrane.

### 4.3. Irradiation

Irradiation was performed using a Gulmay RS225 X-ray machine (Gulmay Ltd., 293 Chertsey, UK) at 200 kVp, 15 mA, and 0.5 mm copper filter.

### 4.4. siRNA Transfection

To silence AKT isoforms, cells were transiently transfected with 50 nM of a pool of siRNAs directed against AKT1 and AKT2. Controls used non-targeting siRNA with lipofectamine 2000. Whole cell lysates were prepared 72 h after transfection to analyze knockdown efficiency by western blotting.

### 4.5. Immunofluorescence Analysis

Immunofluorescence was used to detect γ-H2AX, Rad51 foci, and CENP-F positive cells. Cells were plated on 4-well chamber slides and were allowed to grow for 3 days (based on cell cycle analysis to obtain the highest percentage of G2 cells). Thereafter, cells were exposed to a single dose of 4 Gy of ionizing irradiation either with or without inhibitor treatment for 2 h. Cells were incubated for 6 or 24 h post irradiation and fixed in 3.7% formaldehyde for 10 min. They were then permeabilized with 0.1% Triton X-100 in PBS for 10 min, blocked in 5% bovine serum albumin for 1 h at room temperature, incubated overnight with the primary antibody at 4 °C, washed with PBS, and then incubated in the dark with secondary antibody for 1 h at room temperature. Finally, the cells were washed with PBS and mounted for Vectashield mounting. Imaging was performed with a fluorescence microscope (Axioplan 2, Zeiss, Jena, Germany). For each experimental condition, the number of foci per nucleus (at least 100 nuclei per experiment) were counted and graphed using the Simplot graphics software.

### 4.6. Immunoprecipitation

We performed an immunoprecipitation assay to clarify if Akt is in the complex with BRCA2, RPA, and Rad51. Cells were washed with PBS and lysed with lysis buffer containing 50 mM HEPES, pH 7.4, 150 mM NaCl, 1% NP-40, 0.5%, 10% glycerol, 1 mM EDTA, 1 mM DTT, 1 mM PMSF, 1 mM BGP, and phosphatase as well as protease inhibitors. Next, 2.5 mg protein was obtained from cell lysate and was incubated with 1 µg of antibody (RPA2, Akt1, Akt2, BRCA2, and normal mouse IgG) for 1 h at 4 °C. After adding 50 μL A/G agarose beads, the cell lysates were incubated overnight at 4 °C. The immuno-precipitates were washed three times with washing buffer (10 mM Tris HCL, 150 mM NaCl, 0.5 mM EDTA, phosphatase, and protease inhibitors). After 2 min of centrifugation (2700× *g*), the immuno-complexes that were received were then extracted by boiling in loading buffer for 5 min.

### 4.7. Colony Formation Assay

Colony forming assay were used to check the radiosensitivity of HCT116 cells in different conditions. Sub-confluent cells were plated into 6-well plates at a density of 350 cells/well; 48 h after seeding, the cells were treated with inhibitors for 2 h and irradiated with single doses of 0, 2, and 4 Gy and incubated for 12 days to induce colony formation. Cells were fixed and stained with crystal violet and colonies with more than 50 cells were counted as survivors. The survival fractions were calculated by normalizing the plating efficiency of the treated cells to the plating efficiency of the untreated cells.

## Supplementary Materials

The following are available online at https://www.mdpi.com/1422-0067/20/24/6316/s1.

## Abbreviations

DSBs	double strand breaks
HRR	homologous recombination repair
NHEJ	non homologous end joining
A-NHEJ	alternative non homologous recombination
