# Supplementary material for: Depletion of Akt1 and Akt2 Impairs the Repair of Radiation-Induced DNA Double Strand Breaks via Homologous Recombination
Source: Int J Mol Sci. 2019 Dec 14;20(24):6316. doi: 10.3390/ijms20246316 (PMC6941063; doi:10.3390/ijms20246316)
Supplement: Supplementary file 1 [file ijms-20-06316-s001.pdf]

Supplementary Materials:

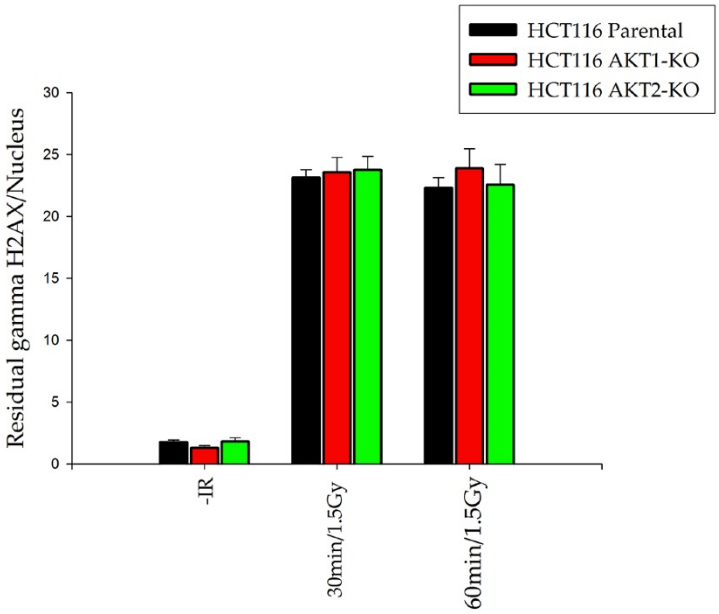

Figure S1. Number of residual gamma H2AX in HCT116 parental, AKT1-KO and AKT2-KO cells. HCT116 parental, AKT1-KO and AKT2-KO cells were irradiated 1.5 Gy and γ-H2AX foci were analyzed 30 min and 60 min after irradiation. Residual γ-H2AX foci were counted in irradiated and non-irradiated cells. The data represent the mean ± SEM of three independent experiments and a total of at least 300 nuclei per condition.

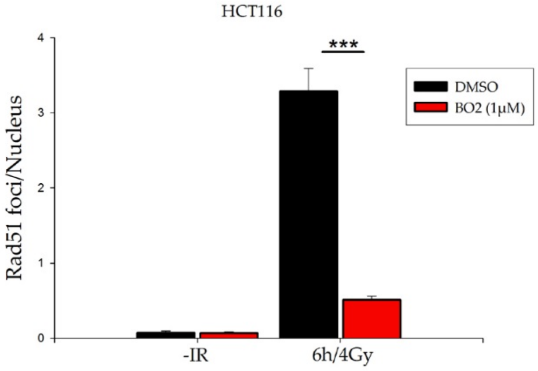

Figure S2. Rad51 foci formation after BO2 treatment. The HCT116 parental cells were treated with 1 μM of BO2 inhibitor for 2 h and irradiated with 4 Gy. The number of Rad%1 foci were counted 6 h after irradiation.

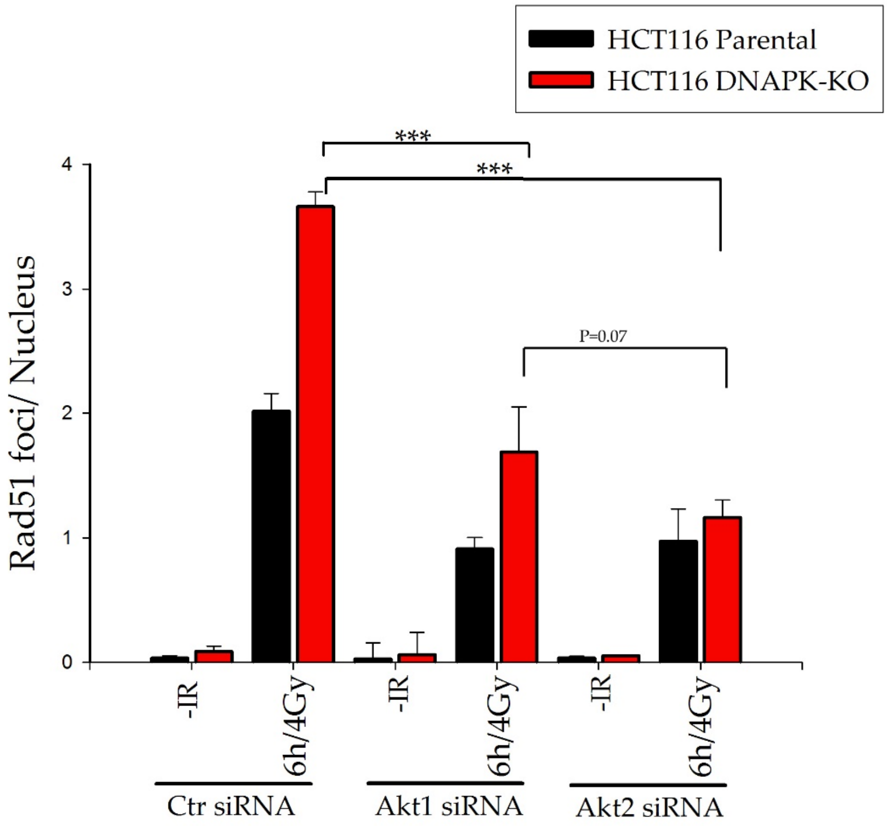

Figure S3. Rad51 foci formation in HCT116 parental and DNAPK-KO cells after AKT1/AKT2 knockdown. HCT116 parental and DNAPK-KO cells were transfected with AKT1-siRNA, AKT2-siRNA, and control-siRNA. The number of Rad51 foci were counted at 6h after 4 Gy. Bars represent the mean number of foci/cell ± SEM from two independent experiments and a total of at least 200 nuclei. (* $p < 0.05$, ** $p < 0.01$, *** $p < 0.001$, Student's t-test).

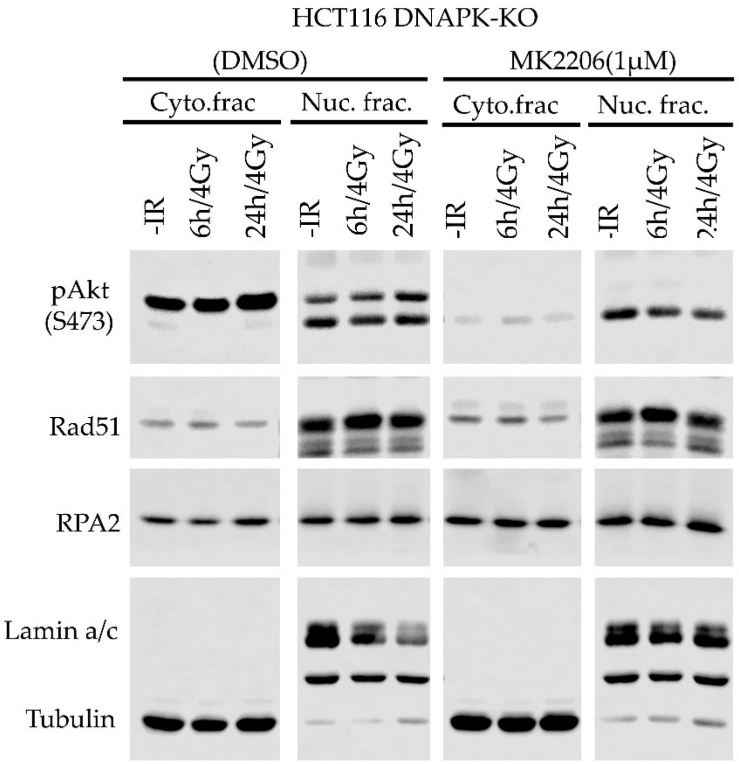

Figure S4. Rad51 and RPA translocation in HCT116 DNAPK-KO cells after MK2206 treatment. MK2206 (1 μM) treated and nontreated HCT116 DNAPK-KO cells were irradiated, and the cytoplasmic and nuclear fractions were prepared 6 and 24 h later.

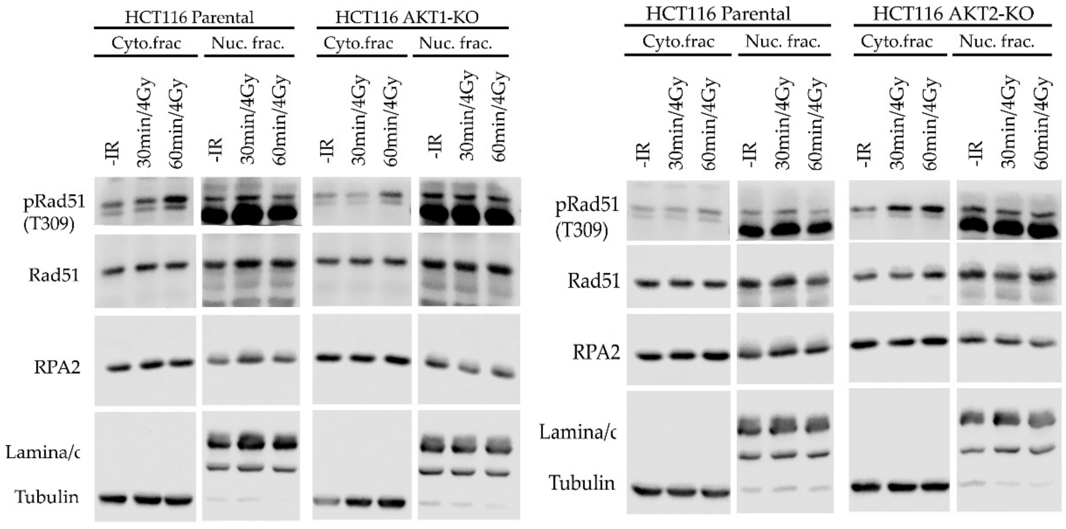

Figure S5. Phosphorylation of Rad51 (T309) after irradiation in AKT1-KO and AKT2-KO cells. Nuclear cytoplasmic fractionations were collected 30 min and 60 min after irradiation with 4 Gy as well as non-irradiated cells.
